# Characterization of Epithelial-Mesenchymal Transition Identifies a Gene Signature for Predicting Clinical Outcomes and Therapeutic Responses in Bladder Cancer

**DOI:** 10.1155/2022/9593039

**Published:** 2022-11-22

**Authors:** Yicun Wang, Hao Zhang, Xiaopeng Hu

**Affiliations:** ^1^Department of Urology, Beijing Chao-Yang Hospital, Capital Medical University, Beijing, China; ^2^Institute of Urology, Capital Medical University, Beijing, China

## Abstract

**Purpose:**

The complex etiological variables and high heterogeneity of bladder cancer (BC) make prognostic prediction challenging. We aimed to develop a robust and promising gene signature using advanced machine learning methods for predicting the prognosis and therapy responses of BC patients.

**Methods:**

The single-sample gene set enrichment analysis (ssGSEA) algorithm and univariable Cox regression were used to identify the primary risk hallmark among the various cancer hallmarks. Machine learning methods were then combined with survival and differential gene expression analyses to construct a novel prognostic signature, which would be validated in two additional independent cohorts. Moreover, relationships between this signature and therapy responses were also identified. Functional enrichment analysis and immune cell estimation were also conducted to provide insights into the potential mechanisms of BC.

**Results:**

Epithelial-mesenchymal transition (EMT) was identified as the primary risk factor for the survival of BC patients (HR=1.43, 95% CI: 1.26-1.63). A novel EMT-related gene signature was constructed and validated in three independent cohorts, showing stable and accurate performance in predicting clinical outcomes. Furthermore, high-risk patients had poor prognoses and multivariable Cox regression analysis revealed this to be an independent risk factor for patient survival. CD8+ T cells, Tregs, and M2 macrophages were found abundantly in the tumor microenvironment of high-risk patients. Moreover, it was anticipated that high-risk patients would be more sensitive to chemotherapeutic drugs, while low-risk patients would benefit more from immunotherapy.

**Conclusions:**

We successfully identified and validated a novel EMT-related gene signature for predicting clinical outcomes and therapy responses in BC patients, which may be useful in clinical practice for risk stratification and individualized treatment.

## 1. Introduction

Bladder cancer (BC) is the most common type of urinary system cancer, with over 570,000 new cases and 210,000 deaths globally in 2020 [1]. Based on the tumor (T) stage, BC patients have been classified into non-muscle-invasive BC (NMIBC) and muscle-invasive BC (MIBC). After transurethral resection of bladder tumor, tumor recurrence and progression were observed in 63% and 11% of NMIBC patients, respectively [2]. Similarly, about half of MIBC patients who underwent radical cystectomy developed local recurrence or distant metastases, and 34% died within a 5-year follow-up period [3]. Individual treatment options for BC patients are currently determined primarily by cancer characteristics such as tumor-node-metastasis (TNM) staging and pathological grade [4]. However, the complex etiological variables and the high heterogeneity BC result in significantly different prognoses, making prognostic prediction difficult [5]. Therefore, a reliable and accurate biomarker in the prognosis of BC and prediction of therapy responses is highly beneficial in directing BC care.

The epithelial-mesenchymal transition (EMT) is a cellular process that allows epithelial cells to acquire mesenchymal characteristics and behaviors with down-regulated epithelial features, most notably the loss of E-cadherin [6, 7]. EMT activation is thought to enhance tumor invasiveness, metastasis, and drug resistance, referring to aggressive tumor type [8, 9]. Several studies demonstrated an association between EMT and progression and survival outcomes in patients with BC [10, 11]. Furthermore, EMT in solid tumors has been shown to correlate with chemotherapy and immunotherapy responses [12–14]. Therefore, EMT-associated characteristics and EMT-based gene signatures have the potential to predict clinical outcomes and responses to chemotherapy and immunotherapy of BC patients.

We identified EMT as a leading risk factor for the survival of BC patients in this study. Advanced machine learning methods were then used to screen prognostic genes, resulting in constructing an EMT-related gene signature validated in multiple cohorts. Moreover, we performed comprehensive analyses of the tumor microenvironment (TME), immune cell infiltration, and therapeutic responses of BC patients to investigate their relationship with EMT and identify potential mechanisms.

## 2. Materials and Methods

### 2.1. The Collection and Pretreatment of Data

Gene expression and relevant clinicopathological data of 405 BC specimens and 19 adjacent normal specimens were obtained from TCGA (https://cancergenome.nih.gov/). After eliminating ineligible samples with overall survival (OS) of less than 30 days, the TCGA dataset including 393 BC patients was used as the training cohort. Subsequently, after screening the GEO database, two datasets were selected for this study based on the following inclusion criteria: (1) histologically confirmed BC samples with gene expression information; (2) samples with complete clinical data; (3) more than 100 samples. The GSE13507 dataset (Illumina human-6 v2.0 expression beadchip) containing 165 BC samples was utilized as validation I cohort. The GSE32894 dataset (umina HumanHT-12 V3.0 expression beadchip) comprising 221 BC samples was utilized as validation II cohort. The detailed information of the above three cohorts is shown in Table 1. All RNA-seq data involved in this study were normalized and log2 transformed.

### 2.2. Study Design

**Figure 1:**
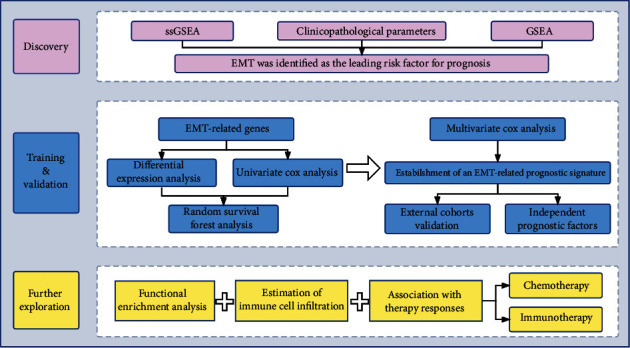
Flowchart of this study. GSEA: gene set enrichment analysis. EMT: epithelial-mesenchymal transition.

As illustrated in Figure 1, three phases including the discovery, training and validation, and further exploration phases were included in this research. In the discovery phase, EMT was identified as the leading risk factor for BC prognosis among various cancer hallmarks. Subsequently, prognostic differentially expressed genes (DEGs) in EMT-related genes were included for random survival forest analysis and stepwise Cox regression to construct a novel EMT-related gene signature, and its prognostic value was also validated in another two independent validation cohorts. Furthermore, we performed functional enrichment analysis, estimation of immune cell infiltration, and therapeutic responses prediction (chemotherapy and immunotherapy) in the above three cohorts.

### 2.3. Identification of the Leading Risk Hallmark for BC Prognosis

Briefly, the single-sample gene set enrichment analysis (ssGSEA) was employed to measure the performance of various confirmed cancer hallmarks in the training cohort using the “gsva” R package. This algorithm was based on gene expression profiles and hallmark annotation gene sets acquired from the Molecular Signatures Database (MSigDB) [15]. Then univariable Cox regression analysis identified EMT with the highest hazard ratio as the leading risk factor for the OS of BC patients and meta-analysis was also applied to compute the pooled hazard ratio of EMT among multiple cohorts to confirm its prognostic role. Moreover, we also applied gene set enrichment analysis (GSEA) to detect the significantly enriched cancer hallmarks in BC samples in comparison with adjacent normal samples using “clusterProfiler” R package [16, 17].

### 2.4. Generation and Verification of the EMT-Related Prognostic Gene Signature

We searched the MSigDB database using the search keyword (Epithelial-mesenchymal transition) and collected 359 EMT-related genes. DEGs between BC specimens and adjacent normal specimens were screened from EMT-related genes using the “limma” R package when the criteria |logFC| > 1 and false discovery rate (FDR < 0.005) were met [18]. With a *p* value threshold of 0.005, we utilized univariable Cox regression analysis to detect prognostic genes among EMT-related genes. Overlapped genes between DEGs and prognostic genes were included for the random survival forest (RSFs) for further selection using the ‘randomForestSRC' R package. The RSFs are an adaptation of random forests for follow-up data analysis, which are tree-based ensemble machine learning algorithms. Feature importance aligned with variable importance measure (VIMP) was applied to select real predictors [19]. Then, the genes identified by RSFs were applied for stepwise Cox regression to construct the EMT-related gene signature using the Akaike information criterion (AIC). Kaplan–Meier and time-independent receiver operating characteristic (ROC) survival assessments using “survminer” and “survivalROC” R packages were applied to comprehensively evaluate the prognostic prediction of EMT-related gene signature in BC prognosis, and two external cohorts GSE13507 and GSE32894 were used for further validation. Lastly, we demonstrated the independent prognostic value of the EMT-related gene signature based on univariable and multivariable Cox regression analysis.

### 2.5. Functional Enrichment Analysis

We utilized the “combat” function from R package “sva” to get rid of the batch effect across GSE13507 and GSE32894 cohorts and merged them into the merged validation cohort. BC patients in both the training and merged validation cohort were classified into high- and low-risk groups based on the optimal cutoff value of EMT-related risk score (ERS) produced by X-tile software. Then, GO and KEGG gene sets were acquired from MSigDB, which were used for the functional annotation of DEGs between risk groups. In the functional enrichment analysis, the input gene list was derived from DEGs between high- and low-risk groups divided by ERS.

### 2.6. TME and Immune Cell Analysis in BC

TME is mainly composed of stromal and immune cells, and it plays a crucial role in tumor prognosis [20]. ESTIMATE algorithm was used to calculate the stromal score, immune score, ESTIMATE score, and tumor purity of each patient using the “estimate” R package [21]. Then, the scores mentioned above were compared between risk groups. Furthermore, the infiltration of various immune cell types in BC was investigated by the CIBERSORT algorithm [22]. To be specific, based on the gene expression feature set of 22 immune cell subtypes, the simulation calculation was performed 1000 times, and the relative composition ratio of the 22 immune cells in each sample was finally obtained. The abundance of 22 immune cell types across risk groups was evaluated and compared.

### 2.7. Evaluation of Therapy Responses

Genomics of Drug Sensitivity in Cancer (GDSC), the largest public pharmacogenomics database, contains gene expression data of many human cancer cell lines and corresponding drug response data [23]. We used the “oncoPredict” package to predict the responses of each patient to various chemotherapeutic drugs based on GDSC [24]. Besides, the IMvigor210 dataset [25] with metastatic urothelial cancer patients treated with antiprogrammed death-ligand (PDL)-1 drug (atezolizumab) and the GSE176307 dataset with metastatic urothelial cancer patients treated with anti-PD-1 or anti-PD-ligand-1 were included in our study. According to gene expression data acquired from the above two datasets, we calculated the ERS of each patient and then divided these patients into high- and low-risk groups. Then, the differences in immunotherapy responses were evaluated.

### 2.8. Statistical Analysis

The D'Agostino and Pearson omnibus normality tests were applied to determine whether each comparison had a normal distribution. Once data met the assumptions of parametric tests, we conducted contrasts using a two-tailed unpaired *t*-test, and the Pearson correlation. The Mann–Whitney *U* test and Spearman correlation were employed when parameters were not normally distributed. Results are considered statistically significant at the level of 5% (*p* < 0.05) except for differential gene expression analyses and univariate Cox regression analysis.

## 3. Results

### 3.1. Identification of EMT as the Leading Risk Factor for Prognosis

**Figure 2:**
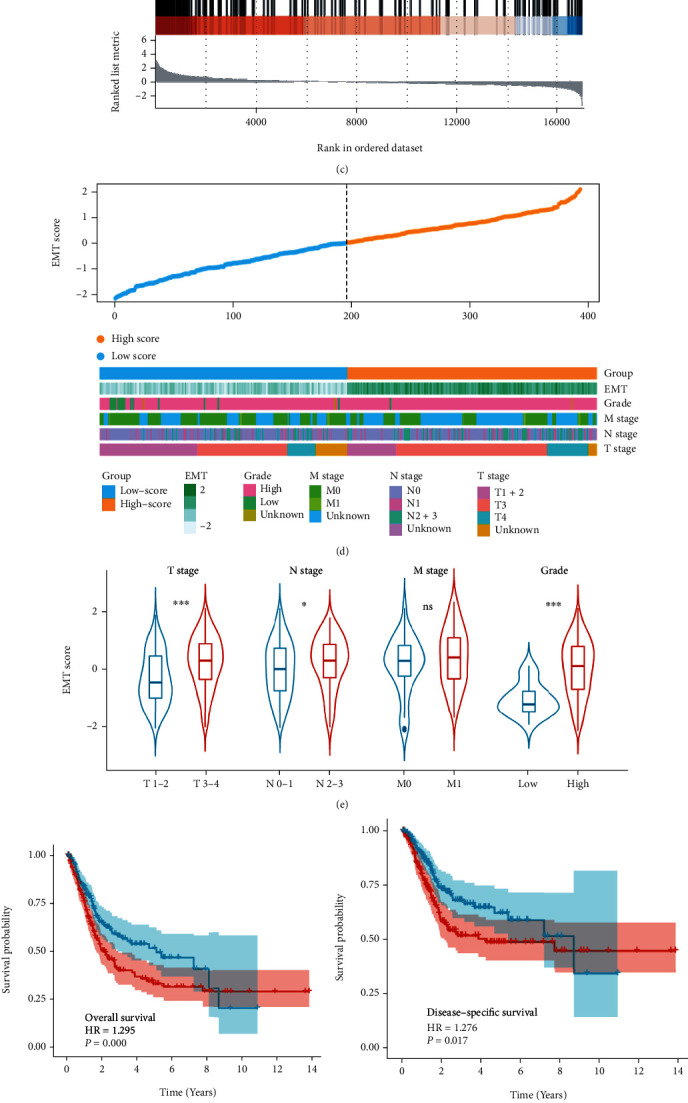
Identification of EMT as the leading risk factor for the prognosis of BC patients. The forest plots show that EMT has the highest HR among various cancer hallmarks in the training cohort (a) and multiple cohorts calculated by meta-analysis (b). (c) GSEA plot illustrates that EMT is significantly enriched in BC samples than adjacent normal samples. (d) The heatmap exhibits the distribution of EMT scores and the patient information of grade, M stage, N stage, and T stage in the training cohort. (e) Violin plot displays that patients with higher T stage, N stage, and pathological grade have higher EMT scores. Kaplan–Meier survival curves depict that high-risk patients divided by EMT scores have worse OS (f), DSS (g), and DFI (h) compared with low-risk patients. HR: hazard ratio. BC: bladder cancer. OS: overall survival. DSS: disease-specific survival. DFI: disease-free interval. ∗*p* < 0.05; ∗∗∗*P* < 0.001; ns, no significance.

In the training cohort, EMT demonstrated a higher HR for overall survival (HR = 1.281, *p* = 0.002) than other cancer hallmarks, which are glycolysis, angiogenesis, etc. (Figure 2(a)). Among all three cohorts, EMT was consistently identified as a risk factor with a pooled HR of 1.43 (Figure 2(b), Supplementary Table 1). GSEA further showed that EMT was significantly annotated in BC patients (Figure 2(c), Supplementary Table 2). Besides, we found that patients in the late clinicopathological stages (tumor stage, node stage, metastasis stage, and pathological grade) had higher ssGSEA scores than patients in the early stage (Figure 2(d)–2(e)). In addition, the Kaplan–Meier survival curves and the log-rank test demonstrated that BC patients with high ssGSEA scores had significantly worse survival outcomes, including OS (HR = 1.295, *p* = 0.009), DSS (disease-specific survival, HR = 1.276, *p* = 0.017), and DFI (disease-free interval, HR = 1.218, *p* = 0.044) (Figure 2(f)–2(h)). All findings mentioned above strongly demonstrated the great influence of EMT on the prognosis of BC patients.

### 3.2. Establishment of the EMT-Related Prognostic Signature

**Figure 3:**
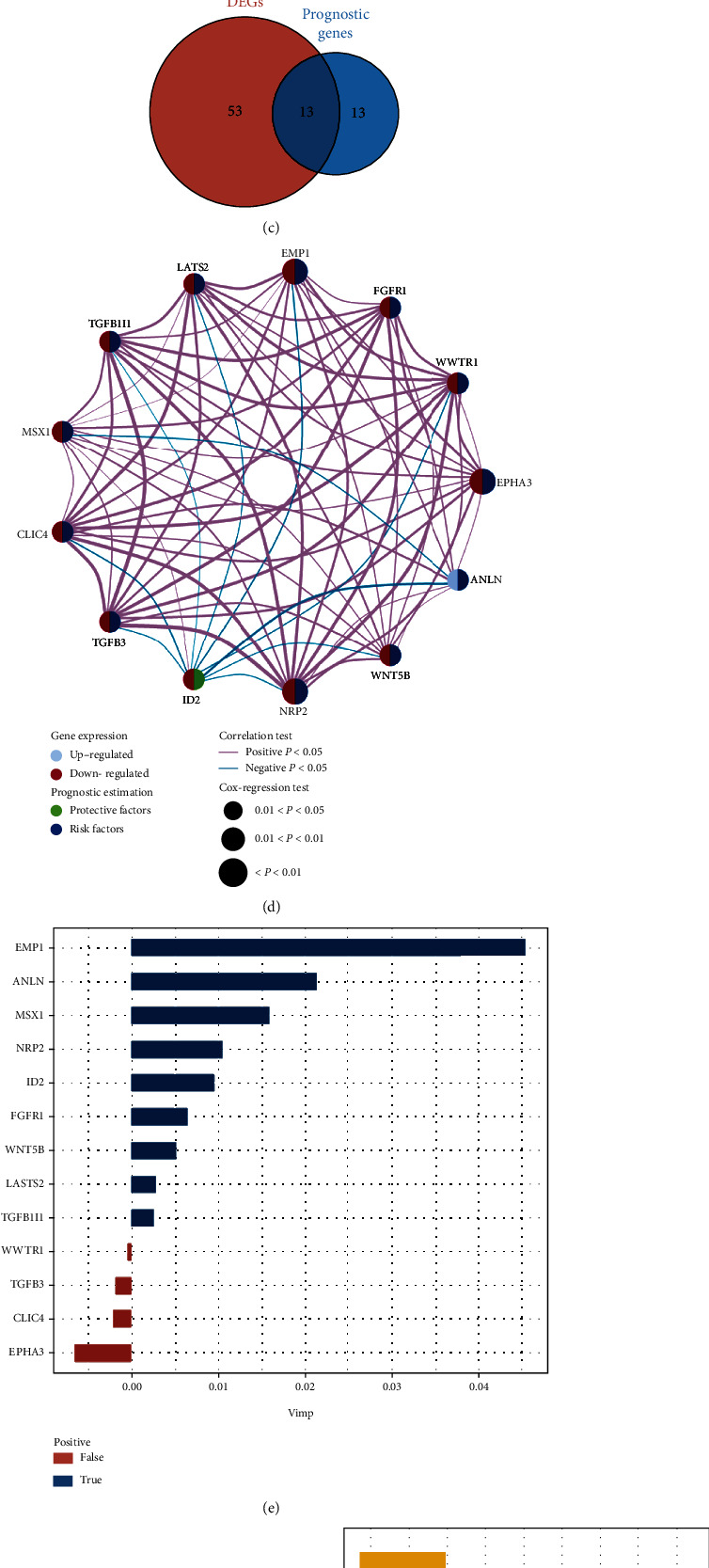
Construction of EMT-related gene signature. Volcano plots show DEGs (a) between BC and adjacent normal samples, and prognostic genes (b) calculated by the univariable Cox regression. Red dots are upregulated genes or risk genes, and blue dots present downregulated genes or protective genes. (c) Venn diagram shows 13 intersected genes between DEGs and prognostic genes. (d) The correlation of 13 EMT-related genes in BC. Upregulated genes and downregulated genes are represented with grey and red colors. Risk genes are described in blue and protective genes are colored in green. The *p* values of the Cox regression test for 13 genes are represented by the size of circles. Correlation analysis is performed on 13 genes, depicted by the connecting lines between each gene. Red and blue lines present positive and negative correlations. (e) Variable importance plot based on random forest survival analysis for 13 genes. Blue color indicates predictive variables, whereas red color represents nonpredictive variables. (f) Forest plot based on univariable Cox regression analysis shows that four genes in this signature are all significantly associated with overall survival. (g) The coefficient of each gene in the gene signature is depicted by bar plots. DEGs: differentially expressed genes.

We acquired EMT-related genes (*n* = 359) from MSigDB for differential gene expression analyses. Then, the intersection of 66 DEGs (13 upregulated and 53 downregulated genes) (Figure 3(a)) and 26 prognostic genes (2 protective and 24 risk genes) (Figure 3(b)) screened 13 candidate genes for further analysis (Figure 3(c)), and the network illustrated a tight relationship among those 13 genes (Figure 3(d)). When all 13 genes were jointly considered by RSF, OS was mainly correlated with 9 genes (*EMP1*, *ANLN*, *MSX1*, *NRP2*, *ID2*, *FGFR1*, *WNT5B*, *LATS2*, and *TGFB1I1*) (Figure 3(e)). Subsequently, these 9 genes were applied to stepwise Cox regression to form the EMT-related gene signature (Figure 3(g)). Among four genes involved in the novel gene signature, three genes (*MSX1*, *ANLN*, and *EMP1*) were risk factors and the remaining one (*ID2*) was a protective factor (Figure 3(f)). The EMT-related signature was calculated as EMT-related risk score = (−0.12119 × *ID*2) + (0.33044 × *MSX*1) + (0.19928 × *ANLN*) + (0.24620 × *EMP*1).

### 3.3. ERS Served as an Independent Prognostic Factor with Promising Value in each Cohort

**Figure 4:**
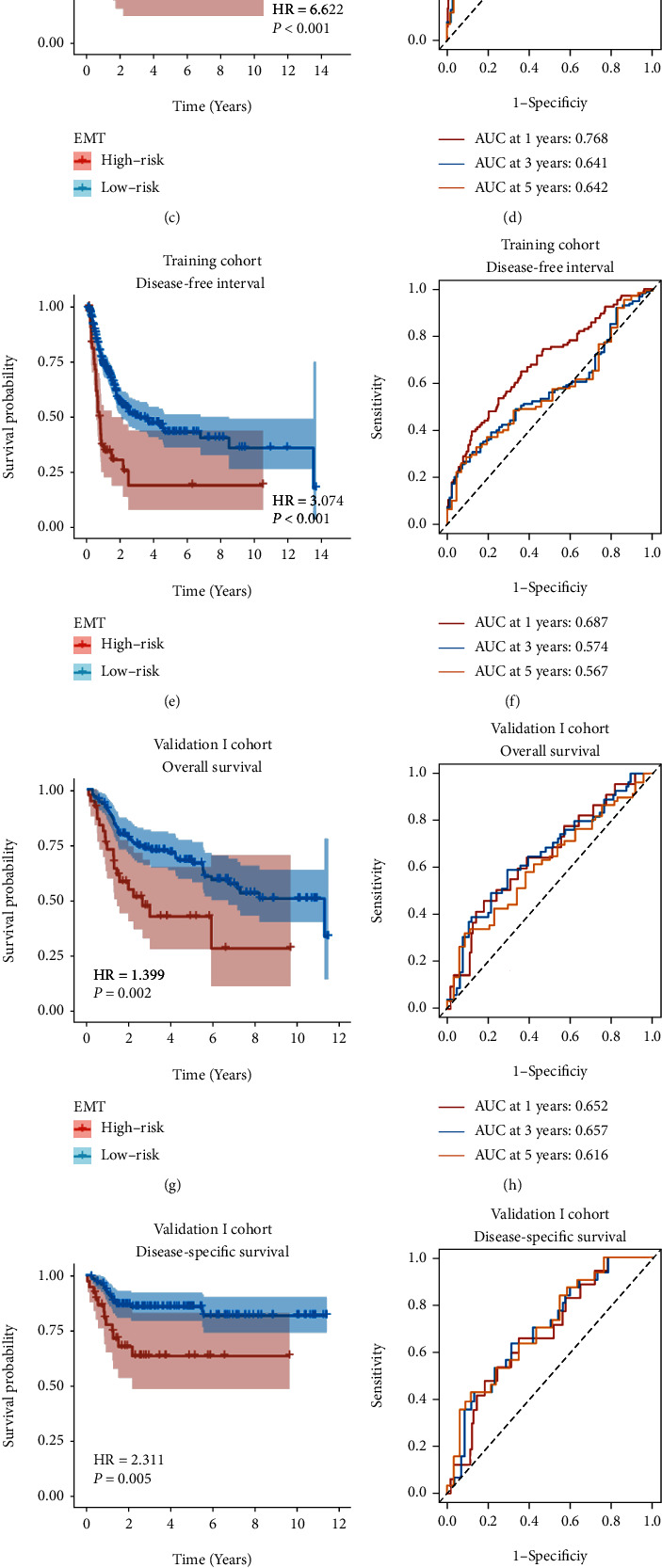
The gene signature serves as a robust and promising predictive factor for survival prediction. Kaplan–Meier survival curves illustrate worse survival outcomes in high-risk patients in the TCGA training cohort (a, c, e), validation I cohort (g, i), and validation II cohort (k). ROC curves for 1-year, 3-year, and 5-year survival prediction depict that this gene signature has a promising and stable predictive performance for BC patients in the training cohort (b, d, f), validation I cohort (h, j), and validation II cohort (l).

Based on the optimal cutoff of ERS value produced by X-tile software, the patients were categorized into high- and low-risk groups in all three training and validation cohorts. Kaplan–Meier curves showed that high-risk patients had significantly lower survival probability in OS (HR = 4.429, *p* < 0.001), DSS (HR = 6.622, *p* < 0.001), and DFI (HR = 3.074, *p* < 0.001) (Figures 4(a), 4(c), and 4(e)). For ERS, the AUC of the predictions for 1, 3, and 5 years was illustrated in Figures 4(b), 4(d), and 4(f). The highest AUC values of OS, DSS, and DSI were 0.686, 0.768, and 0.687. In the validation I cohort, the survival benefits of low-risk patients were significantly better than low-risk patients (OS: HR = 1.399, *p* = 0.002; DSS: HR = 2.311, *p* = 0.005) (Figures 4(g) and 4(i)). The maximum AUC values of OS and DSS in the validation I set were 0.657 and 0.703 (Figures 4(h) and 4(j)). Similarly, ERS also performed well in prognostic prediction in the validation II cohort (HR = 10.606, *p* < 0.001, highest AUC = 0.821) (Figures 4(k) and 4(l)). Overall, ERS was accurate and robust in evaluating the prognosis of BC patients.

In univariable Cox regression analysis, the T stage, N stage, M stage, and ERS were significantly correlated with OS, while ERS was demonstrated as the only independent prognostic factor for OS by multivariable Cox regression analysis (HR = 3.831, *p* < 0.001) in the training cohort (Table 2). Similar results can also be obtained in two validation cohorts (Supplementary Table 3-5).

### 3.4. Functional Enrichment and Immune Cell Infiltration Analyses

**Figure 5:**
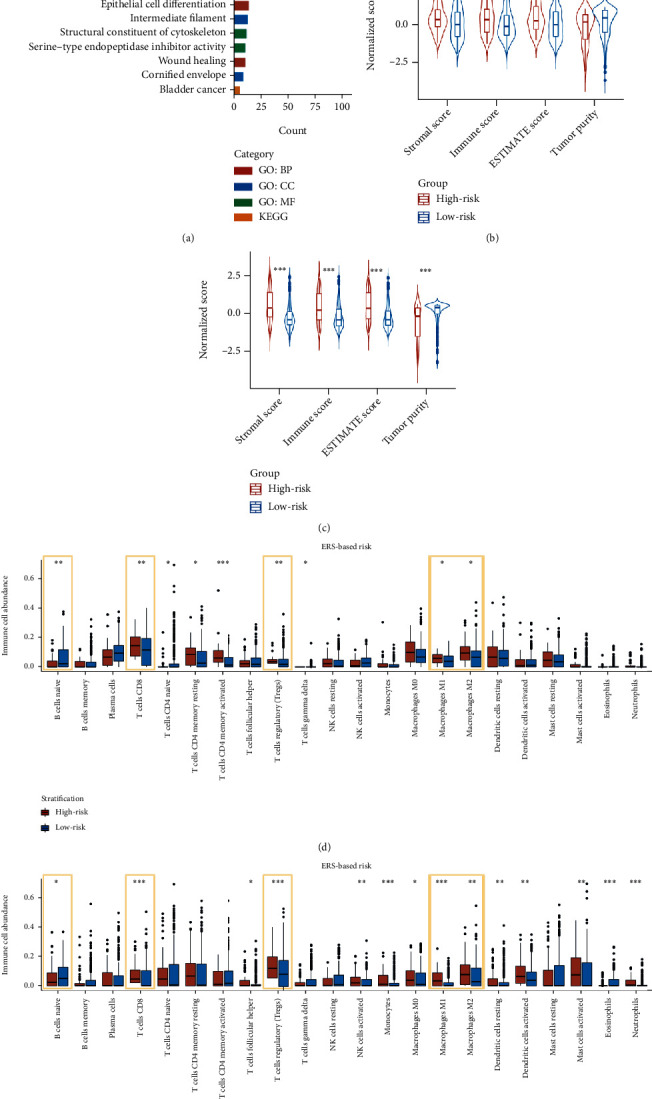
Functional enrichment and immune cell infiltration analyses based on the gene signature. (a) Bar graph displays significantly enriched pathways in high-risk patients. Violin plots show higher immune score, stromal score, ESTIMATE score, and lower tumor purity in the high-risk patients compared with low-risk patients in the training (b) and merged validation cohorts (c). Box plots depict that CD8+ T cells, Tregs, M1 macrophages, and M2 macrophages are higher infiltrated and B naïve cells are lower infiltrated and in high-risk patients in both training (d) and merged validation (e) cohorts. ∗*p* < 0.05; ∗∗*p* < 0.01; ∗∗∗*p* < 0.001.

According to functional enrichment analysis, DEGs between risk groups exhibited significant enrichment in EMT-associated pathways, including extracellular exosomes, epithelial cell differentiation, and wound healing (Figure 5(a)). Besides, as shown in Supplementary Figure 1, the EMT scores representing the performance of EMT process were consistently correlated with expression levels of four genes and ERS (*R* = 0.59, *p* < 0.0001). Moreover, the comparison of TME components showed that high-risk patients had significantly greater stromal scores (*p* < 0.05), immune scores (*p* < 0.01), ESTIMATE scores (*p* < 0.05), and lower tumor purity (*p* < 0.01) than the low-risk patients (Figure 5(b)). Similar results can also be observed in the merged validation cohort (Figure 5(c)). Besides, by analyzing the infiltration of immune cells, we found that CD8+ T cells, Tregs, Macrophages M1, and Macrophages M2 were higher infiltrated, and B naïve cells were lower infiltrated in the TME of high-risk patients in both training and merged validation cohorts (Figures 5(d) and 5(e)).

### 3.5. Role of ERS in Predicting Chemotherapeutic Sensitivity and Immunotherapeutic Response

**Figure 6:**
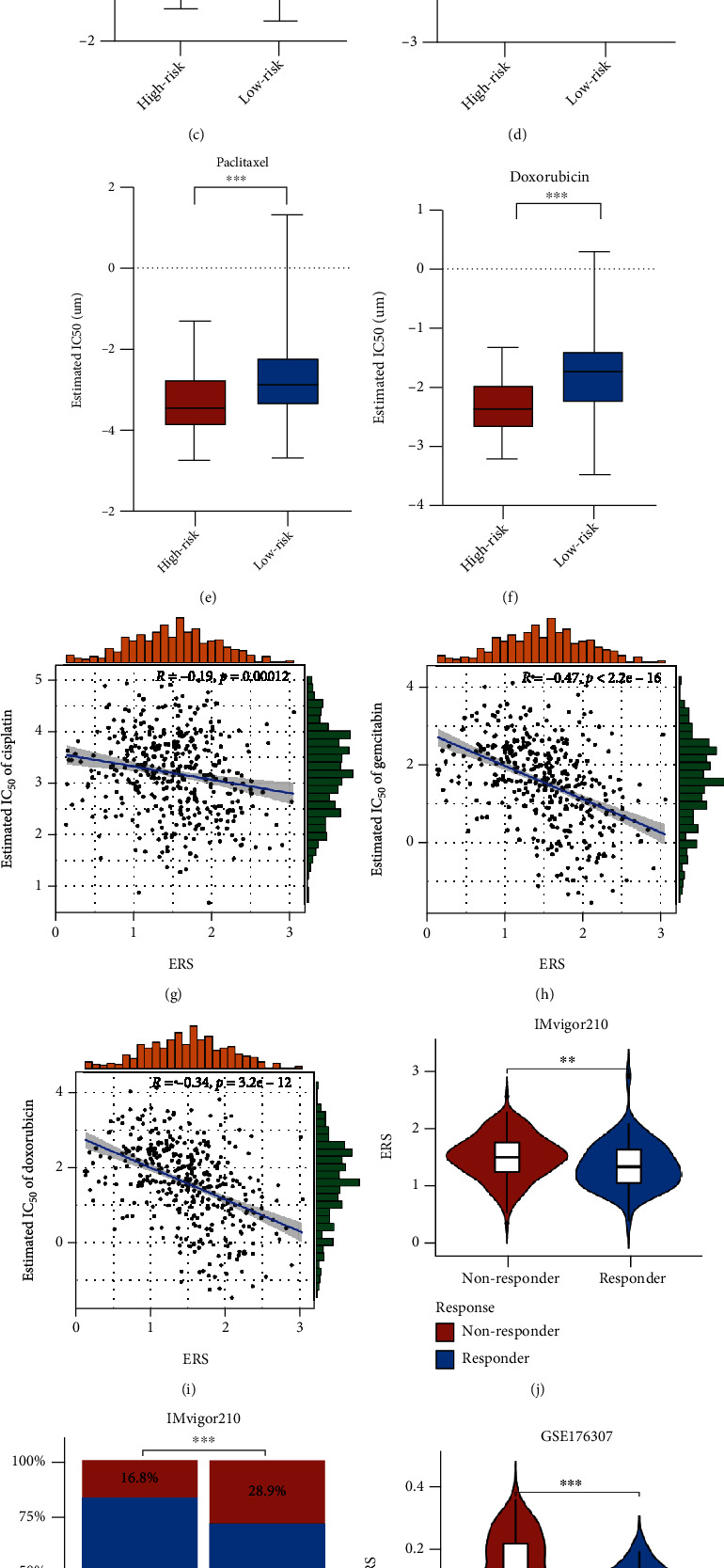
EMT-related gene signature predicts chemotherapeutic and immunotherapeutic benefits. Box plots display lower IC_50_ values of six commonly used chemical drugs in high-risk patients (a-f). Scatter plots illustrate negative correlations between ERS and the estimated IC50 values of cisplatin (g), gemcitabine (h), and doxorubicin (i). Violin plots show that ERS of nonresponders is significantly higher than that of responders in the immunotherapy cohort of IMvigor210 (j) and GSE176307 (l). High-risk patients present significantly lower percentages of responses (CR/PR) and higher percentages of nonresponses (SD/PD) in both IMvigor210 (k) and GSE176307 (m). IC_50_: half-maximal inhibitory concentration. ERS: EMT-related risk score. CR: complete response. PR: partial response. SD: stable disease. PD: progressive disease. ∗*p* < 0.05; ∗∗*p* < 0.01; ∗∗∗*p* < 0.001.

Chemotherapies are extensively used for BC treatment in clinical practice. Therefore, we estimated the therapy responses of each patient to six commonly used drugs (Cisplatin, Vinblastine, Gemcitabine, Methotrexate, Paclitaxel, and Doxorubicin) by evaluating their IC50 values based on the GDSC database. As a result, the IC50 values of Cisplatin (*p* < 0.001), Vinblastine (*p* < 0.001), Gemcitabine (*p* < 0.001), Methotrexate (*p* < 0.05), Paclitaxel (*p* < 0.001), and Doxorubicin (*p* < 0.001) in the high-risk group was significantly lower than that in the low-risk group (Figures 6(a)–6(f)). Moreover, the results of correlation analysis also showed that ERS was negatively related with the IC50 values of Cisplatin (*r* = −0.19, *p* = 0.00012), Gemcitabine (*r* = −0.47, *p* < 2.2*e* − 16), and Doxorubicin (*r* = −0.34, *p* < 3.2*e* − 12) (Figure 6(g)–6(i)). These results indicated that patients with higher ERS are more sensitive to chemotherapy.

Anti-PD1/PDL1 drugs were currently approved by the FDA for the treatment of BC, with 3 anti-PDL-1 drugs (atezolizumab, durvalumab, and avelumab), and 2 anti-PD-1 drugs (nivolumab and pembrolizumab). Thus, we evaluated whether the ERS might be utilized for the prediction of therapy responses to immunological checkpoint blockade (ICB) treatment based on the above two cohorts. As a result, responders displayed lower ERS compared with non-responders in both IMvigor210 (*p* < 0.01, Figure 6(j)) and GSE176307 (*p* < 0.001, Figure 6(l)). Furthermore, through allocating patients into high- and low-risk groups based on ERS, we found that high-risk patients had significantly lower percentages of responses (complete response, CR/partial response, PR) and higher percentages of nonresponses (stable disease, SD/progressive disease, PD) in both IMvigor210 (*p* < 0.001, Figure 6(k)) and GSE176307 (*p* < 0.001, Figure 6(m)) cohorts.

## 4. Discussion

EMT is the process by which epithelial cells transform into mesenchymal-like cells with decreased expression of epithelial markers, such as E-cadherin, and upregulation of mesenchymal markers expressions [7, 26, 27]. Decreased expression of epithelial markers in BC patients was correlated with disease progression (higher grade and stage), and EMT-related molecules (*β*-catenin or plakoglobin) were associated with poor DSS [9]. Sayan et al. found a link between EMT expression regulator Zeb-1 and enhanced urothelial cancer cell invasion and migration [11]. Furthermore, there was an association between EMT levels in solid tumors and their responses to chemotherapy and immunotherapy [12–14]. The above studies suggested that EMT has significant prognostic and therapeutic potential in solid tumors. However, there is a lack of EMT-related gene signatures for predicting prognosis and therapeutic response in BC.

We developed an EMT-related gene signature for predicting survival outcomes (OS, DSS, and DFI) of BC patients. The robustness and applicability of this gene signature were verified by two independent cohorts from two different RNA-seq platforms. Our findings indicated that patients designated as high-risk using the novel signature had poor survival outcomes than low-risk patients. Furthermore, multivariable Cox analysis revealed that the gene signature was the only independent predictor of OS after adjusting for clinical factors. No gene overlap was observed between these two signatures, in contrast to a previous study that used seven EMT-linked genes to predict the prognosis of patients with MIBC [28]. Our gene signature with only four genes performed well across all three cohorts, and the AUC values were higher than the previous one. Moreover, we comprehensively analyzed the relationship between EMT and chemotherapeutic and immunotherapeutic responses, providing the foundation for further research.

Functional enrichment analyses were performed to better understand the potential mechanisms of different clinical outcomes among patients. Our results revealed that DEGs between these two groups were significantly enriched in extracellular exosomes, cell proliferation, epithelial cell differentiation, wound healing, BC, and other variables. Shan et al. demonstrated that exosomes produced by cancer-associated fibroblasts might induce metastasis of BC cells by increasing their EMT [29]. Wang et al. reported that increased UCA1 expression in BC-derived exosomes promotes tumor growth via EMT [30]. McConkey et al. proposed that EMT is essential for the cell proliferation required for wound healing [31]. Moreover, the expression levels of four genes and ERS were significantly correlated with the EMT score, indicating that the novel signature was associated with the EMT process in BC. Our findings indicate that EMT-related characteristics may be closely associated with the development and progression of BC, and the EMT-related gene signature has great potential for prognostic gene-function-based prediction.

TME plays a vital role in cancer formation and treatment resistance [20]. TME dysregulation promotes BC progression and metastasis [32]. This study reported that high-risk patients had a higher immune score, stromal score, and lower tumor purity, suggesting a potential role of ERS in TME and was consistent with previous research [33]. The prognostic and predictive potential of immune cell infiltration in BC have been investigated, and several immunological markers have been linked with treatment outcomes [34]. Some studies reported that higher CD8+ T cell infiltration in the epithelium and invasive margin indicated a longer OS or DSI in BC patients [35–37]. However, one study reported a negative correlation between stromal CD8+ cell infiltration and survival outcomes [38]. CD8+ T cells were found to be more infiltrated in the tumors of high-risk patients, suggesting that we should pay more attention to this interesting phenomenon. Tumor-infiltrating Tregs are important suppressors of antitumor immunity. A meta-analysis study showed that Tregs were associated with poor OS in many solid tumors, consistent with our results [39]. Macrophages are essential components of innate immunity and can be classified into proinflammatory macrophages (M1) and anti-inflammatory macrophages (M2). In a previous study, the higher density of M2 macrophages was associated with higher pathological and histological grades in BC patients [40, 41]. Furthermore, there was a tendency for patients with high macrophage levels to have poor survival [34]. Our results revealed that M2 macrophage infiltration was significantly higher in high-risk patients, consistent with previous research.

We selected six representative chemical drugs and found that high-risk patients were more sensitive to these drugs. Negative correlations between ERS and drug sensitivity were found in three chemotherapeutic drugs, including gemcitabine, cisplatin, and doxorubicin. The above results revealed that high-risk patients divided by ERS might be more likely to benefit from chemotherapy. Immune checkpoint inhibitors were approved for clinical use in metastatic BC in 2017. Unfortunately, only 21.1% of metastatic BC patients responded to ICB treatment (pembrolizumab) [42]. Therefore, predictive biomarkers are required to identify a specific subset of patients who may respond to immunotherapy. Our analysis indicated that low-risk patients showed a better response in two cohorts, suggesting that our model may be useful for identifying patients who may benefit from immunotherapy. These findings indicate that the gene signature can potentially guide clinical treatment decisions regarding chemotherapy and immunotherapy.

Although this is an original signature with promising clinical applications, this study has some limitations. Despite the robust performance of our gene signature in prognostic prediction, more prospective studies with larger sample sizes are required to validate its general application. Moreover, an interesting phenomenon observed in this study, particularly regarding the underlying mechanisms of biological functions and immune cell infiltration, requires further experimental investigation. Furthermore, the lack of experimental and clinical evidence for verifying drug responses is also a limitation that should be addressed in the future.

## 5. Conclusion

In summary, we identified and verified a novel EMT-related gene signature with high prognostic prediction efficacy across multiple independent cohorts. Moreover, it was associated with the chemotherapeutic and immunotherapeutic responses of BC patients. This novel signature had great potential for predicting prognosis and guiding clinical therapies.

## Figures and Tables

**Table 1:** Clinical characteristics of BC patients in three independent cohorts.

Characteristics	TCGA	GSE13507	GSE32894	Overall
Total	393	165	221	779
Application	Training	Validation I	Validation II	
Age (%)				
<60	85 (21.6)	42 (25.5)	38 (17.2)	165 (21.2)
≥ 60	308 (78.4)	123 (74.5)	183 (82.8)	614 (78.8)
Sex (%)				
Female	103 (26.2)	30 (18.2)	60 (27.1)	193 (24.8)
Male	290 (73.8)	135 (81.8)	161 (72.9)	586 (75.2)
T stage (%)				
Ta		24 (14.5)	109 (49.3)	133 (17.1)
T1	3 (0.8)	80 (48.5)	61 (27.6)	144 (18.5)
T2	113 (28.8)	31 (18.8)	43 (19.5)	187 (24.0)
T3	190 (48.3)	19 (11.5)	7 (3.2)	216 (27.8)
T4	54 (13.7)	11 (6.7)	1 (0.5)	66 (8.5)
Unknown	33 (8.4)			33 (4.2)
N stage (%)				
N0	227 (57.8)	149 (90.3)		376 (67.4)
N1	44 (11.2)	8 (4.8)		52 (9.3)
N2	74 (18.8)	6 (3.6)		80 (14.3)
N3	7 (1.8)	1 (0.6)		8 (1.4)
Unknown	41 (10.4)	1 (0.6)		45 (8.1)
M stage (%)				
M0	188 (47.8)	158 (95.8)		346 (62.0)
M1	10 (2.5)	7 (4.2)		17 (3.0)
Unknown	195 (49.6)			195 (34.9)
Grade (%)				
High	372 (94.7)	60 (36.4)	91 (41.2)	523 (67.1)
Low	18 (4.6)	105 (63.6)	128 (57.9)	251 (32.2)
Unknown	3 (0.8)		2 (0.9)	5 (0.6)
Clinical outcomes				
Overall survival (%)				
Alive	219 (55.7)	96 (58.2)	196 (88.7)	511 (65.6)
Deceased	174 (44.3)	69 (41.8)	25 (11.3)	268 (34.4)
Cancer-specific (%)				
Alive	274 (69.7)	133 (80.6)		407 (72.9)
Deceased	119 (30.3)	32 (19.4)		151 (27.1)
Progression-free (%)				
Censored	222 (56.5)			222 (56.5)
Event	171 (43.5)			171 (43.5)
Follow-up time (months, mean ± SD)	27.81 (27.93)	48.38 (37.70)	40.44 (25.33)	35.75 (30.74)

**Table 2:** Univariate and multivariate Cox regression analysis of clinical characteristics and ERS with overall survival in TCGA cohort.

Variables	No. of Patients	Univariate			Multivariate		
		HR	95% CI	*p* value	HR	95% CI	*p* value
Age	162	1.023	0.997–1.049	0.078			
Gender	162						
Female	36	Reference					
Male	126	0.61	0.363–1.022	0.061			
T stage	162						
T_1+2_	53	Reference					
T_3_	87	2.27	1.167–4.416	0.016	1.669	0.842–3.311	0.142
T_4_	22	3.138	1.419–6.941	0.005	2.133	0.916–4.965	0.079
N stage	162						
N_0_	112	Reference					
N_1_	21	1.99	1.033–3.836	0.04	1.364	0.676–2.754	0.386
N_2+3_	29	2.745	1.582–4.761	< 0.001	1.627	0.855–3.097	0.138
M stage	162						
M_0_	155	Reference					
M_1_	7	2.532	1.01–6.346	0.048	1.354	0.492–3.725	0.558
Grade	162						
High	147	Reference					
Low	15	0.272	0.037–1.979	0.199			
ERS	162	4.429	2.534–7.741	< 0.001	3.831	2.138–6.865	< 0.001

## Data Availability

The data used to support the findings of this study are available from the corresponding author upon request.

## Supplementary Materials

Supplementary Materials Supplementary Table 1. Univariate Cox analysis based on ssGSEA scores of cancer hallmarks in all three cohorts. Supplementary Table 2. GSEA of cancer hallmarks. Supplementary Table 3. Univariate and multivariate Cox regression analysis of clinical characteristics and ERS with survival outcomes (DSS, DFI) in the TCGA cohort. Supplementary Table 4. Univariate and multivariate Cox regression analysis of clinical characteristics and ERS with survival outcomes (OS, DSS) in the GSE13507 cohort. Supplementary Table 5. Univariate and multivariate Cox regression analysis of clinical characteristics and ERS with OS in the GSE32894 cohort. Supplementary Figure 1. Correlations between the EMT score and ERS, and expression levels of four genes included in the novel signature.
