# Supplementary material for: Characterization of Epithelial-Mesenchymal Transition Identifies a Gene Signature for Predicting Clinical Outcomes and Therapeutic Responses in Bladder Cancer
Source: Dis Markers. 2022 Nov 22;2022:9593039. doi: 10.1155/2022/9593039 (PMC9708359; doi:10.1155/2022/9593039)
Supplement: Supplementary Materials — Supplementary Table 1. Univariate Cox analysis based on ssGSEA scores of cancer hallmarks in all three cohorts. Supplementary Table 2. GSEA of cancer hallmarks. Supplementary Table 3. Univariate and multivariate Cox regression analysis of clinical characteristics and ERS with survival outcomes (DSS, DFI) in the TCGA cohort. Supplementary Table 4. Univariate and multivariate Cox regression analysis of clinical characteristics and ERS with survival outcomes (OS, DSS) in the GSE13507 cohort. Supplementary Table 5. Univariate and multivariate Cox regression analysis of clinical characteristics and ERS with OS in the GSE32894 cohort. Supplementary Figure 1. Correlations between the EMT score and ERS, and expression levels of four genes included in the novel signature. [file 9593039.f1.zip › Supplementary Table 4.docx]

Supplementary Table 3: Univariate and multivariate Cox regression analysis of clinical characteristics and ERS with survival outcomes (OS, DSS) in the GSE13057 cohort

GSE13507 OS

| Variables | Univariate | | | Multivariate | | |
| --- | --- | --- | --- | --- | --- | --- |
|  | HR | 95% CI | *P-*value | HR | 95% CI | *P-*value |
| Age | 1.071 | 1.045—1.097 | ＜0.001 | 1.081 | 1.052--1.111 | ＜0.001 |
| Gender |  |  |  |  |  |  |
| Female | Reference | |  |  |  |  |
| Male | 0.629 | 0.354—1.118 | 0.114 |  |  |  |
| T stage |  |  |  |  |  |  |
| T1 | Reference | |  |  |  |  |
| T2+3+4 | 5.936 | 2.343—15.038 | ＜0.001 | 6.43 | 2.435--16.982 | ＜0.001 |
| Ta | 0.365 | 0.188—0.708 | 0.003 | 0.563 | 0.264--1.204 | 0.139 |
| N stage |  |  |  |  |  |  |
| N0 | Reference | |  |  |  |  |
| N1 | 6.865 | 3.033—15.537 | ＜0.001 | 2.091 | 0.773--5.658 | 0.146 |
| N2+3 | 8.149 | 3.115—21.316 | ＜0.001 | 6.09 | 1.706--21.745 | 0.005 |
| M stage |  |  |  |  |  |  |
| M0 | Reference |  |  |  |  |  |
| M1 | 10.246 | 4.522—23.212 | ＜0.001 | 3.337 | 1.136--9.801 | 0.028 |
| Grade |  |  |  |  |  |  |
| High | Reference |  |  |  |  |  |
| Low | 0.373 | 0.23—0.606 | ＜0.001 | 0.658 | 0.36--1.202 | 0.174 |
| ERS | 1.399 | 1.083—1.806 | 0.01 | 0.852 | 0.48--1.511 | 0.583 |

GSE13507 DSS

| Variables | Univariate | | | Multivariate | | |
| --- | --- | --- | --- | --- | --- | --- |
|  | HR | 95% CI | *P-*value | HR | 95% CI | *P-*value |
| Age | 1.053 | 1.017—1.09 | 0.004 | 1.06 | 1.02—1.11 | 0.006 |
| Gender |  |  |  |  |  |  |
| Female | Reference | |  |  |  |  |
| Male | 0.456 | 0.21—0.992 | 0.048 | 0.73 | 0.31—1.72 | 0.4725 |
| T stage |  |  |  |  |  |  |
| T1 | Reference | |  |  |  |  |
| T2+3+4 | 3.932 | 1.501—10.3 | 0.005 | 4.32 | 1.54—12.08 | 0.0053 |
| Ta | 0.117 | 0.05—0.273 | ＜0.001 | 0.16 | 0.06—0.43 | ＜0.001 |
| N stage |  |  |  |  |  |  |
| N0 | Reference | |  |  |  |  |
| N1 | 13.709 | 5.621—33.434 | ＜0.001 | 2.76 | 0.86—8.88 | 0.0886 |
| N2+3 | 14.678 | 4.184—51.491 | ＜0.001 | 2.36 | 0.42—13.2 | 0.3286 |
| M stage |  |  |  |  |  |  |
| M0 | Reference |  |  |  |  |  |
| M1 | 13.863 | 5.552—34.616 | ＜0.001 | 6.53 | 1.76—24.28 | 0.0051 |
| Grade |  |  |  |  |  |  |
| High | Reference |  |  |  |  |  |
| Low | 0.173 | 0.079—0.377 | ＜0.001 | 0.36 | 0.14—0.93 | 0.0349 |
| ERS | 2.311 | 1.279—4.177 | 0.006 | 0.51 | 0.19—1.35 | 0.1738 |
